# Transformative capacity and local action for urban sustainability

**DOI:** 10.1007/s13280-018-1086-z

**Published:** 2018-09-11

**Authors:** Vanesa Castán Broto, Gregory Trencher, Ewa Iwaszuk, Linda Westman

**Affiliations:** 1 Interdisciplinary Centre for the Social Sciences, Urban Institute and Department of Geography, University of Sheffield, 219 Portobello, Sheffield, S2 4DP UK; 2 Graduate School of Environmental Studies, Tohoku University, 468-1 Aramaki-aza-Aoba, Aobaku, Sendai, 980-0845 Japan; 3 Ecologic Institute, Pfalzburger Str. 43-44, 10717 Berlin, Germany; 4 Department of Geography and Environmental Management, University of Waterloo, 200 University Avenue West, Waterloo, ON N2L 3G1 Canada

**Keywords:** Cities, Evaluation, Transformative capacity, Urban sustainability, Urban transformations

## Abstract

There is a consensus about the strategic importance of cities and urban areas for achieving a global transformation towards sustainability. While there is mounting interest in the types of qualities that increase the capacity of urban systems to attain deep transformations, empirical evidence about the extent to which existing institutional and material systems exhibit transformative capacity is lacking. This paper thereby seeks to determine the extent to which sustainability initiatives led by local governments and their partners reflect the various components that the literature claims can influence the emergence of transformative capacity as a systemic property of urban settings. Using an evaluative framework consisting of ten components of transformative capacity and associated indicators, the specific objective is to identify patterns in these initiatives regarding the presence of individual components of transformative capacity and their interrelations with other components. The analysis of 400 sustainability initiatives reveals thin evidence of transformative capacity. When detected, evidence of transformative capacity tended to emerge in relation to wider processes of institutional- and social-learning and initiatives that linked outcomes to a city-wide vision of planning and development. However, instances of such initiatives were rare. This widespread lack of evidence for transformative capacity raises concerns that this set of attributes normalised in the literature is in fact rarely found in sustainability action on the ground.

## Introduction

Since the inclusion of an explicitly urban-focused objective among the 17 United Nations Sustainable Development Goals adopted in 2015, urban settlements are receiving increasing attention in policy and political discourse around sustainable development (Barnett and Parnell 2016). Given that cities and urban areas present multiple and significant opportunities to translate global and national objectives into action (Hoornweg et al. 2011; UN-Habitat 2011; IPCC 2014), this discourse emphasises that urbanisation processes must make a contribution towards the global imperative of carrying out societal transformations towards greater sustainability (Parnell 2016). This focus on urban areas resonates with the sustainability transitions literature that seeks to understand and spur possibilities for achieving widespread change through various forms of experimentation in urban settings (Bulkeley et al. 2010; Frantzeskaki et al. 2016; Luederitz et al. 2017). The experimentation paradigm has attracted widespread interest from scholars, policymakers and societal practitioners and it is driven by awareness that current ways of organising urban systems are unsustainable, and that novel and often radically different forms of social or technological innovation are required (Marvin et al. 2018). Initiatives seeking to tackle urban sustainability issues are often characterised by an ethos of experimentation (Bulkeley and Castán Broto 2013). Experimentation implies pioneering and breaking with convention, but urban sustainability experiments are often criticised because of their limited scale, and the difficulties to extract lessons from experiments that can be applied elsewhere (Evans and Karvonen 2014).

Renewed attention to cities and urban areas as sites of transformation poses significant governance challenges (Simon and Leck 2015) since the urban transformation agenda demands consideration of the structural changes required to reconfigure interlinked environmental, social and economic relations (McCormick et al. 2013a, b) and bring about fundamental changes to break away from unsustainable technologies, practices and ways of organising society (Wolfram et al. 2016; Geels et al. 2017). Yet cities are not isolated entities but are embedded in regional and global networks both in terms of material (Grimm et al. 2008) and information flows (Acuto 2016). Thus, urban transformations must involve trans-local, cross-scalar changes within and beyond administrative boundaries. A fundamental question is whether current urban systems can deliver such transformative change.

In this paper, we focus on the ability to develop processes and institutions to drive radical change in urban settings towards more sustainable futures. Wolfram (2016) defines urban transformative capacity as the ability of an urban system (inclusive of physical and human dimensions) to reconfigure and move towards a new and more sustainable state. This conception of urban transformative capacity is founded on the identification of particular attributes that influence the extent to which transformative capacity might emerge as a systemic property in interlinked human and material systems in an urban setting (ibid). In the context of urban governance, the notion of transformative capacity can thus serve as an indicator or predictor of the ability of a dynamic constellation of public and private actors to steer urban development in a radically different direction from historical pathways. Urban transformative capacity also relates to the engagement of such actors with existing institutions and processes of institutionalisation that can deliver such radical change (Castán Broto et al. 2015). This definition aligns with socio-ecological understandings of transformative capacity, which emphasise such capacity as part of adaptive socio-ecological relations (Boyd and Folke 2011; O’Brien 2012).

Cities constitute complex socio-ecological systems in which activities on multiple scales interact to create development towards transformative change (Folke et al. 2010). Capacity to create ‘untried beginnings’ for socio-ecological systems (Westley et al. 2011) derives from aspects such as the ability to foster knowledge-building, organisational learning and polycentric, multiactor decision making processes (Folke et al. 2005). Such capacities also depend on the dynamics of knowledge production and the extent to which there is room to challenge dominant power relations (Cote and Nightingale 2012).

The concept of transformative capacity focuses on institutions and processual aspects that can bring about sustainability transformations. Transformation is not limited to a simple linear process of achieving desired futures by facilitating incremental change or by reverse-engineering (i.e. scenario or back-casting guided). Instead, it is about fostering a cultural transformation, an ongoing process of social learning through which sustainability objectives are seen as a ‘moving target’ and never truly accomplished. Contemporary societies focus on maintaining system functions; that is, coping and progressively adapting to partial change. As such, experiences of deliberate and quick transformations in human societies are rare (Patt 2010).

There is a consensus about the vital role that local governments can play in advancing urban sustainability through policy and in creating the institutional conditions to foster social and technical innovation across different societal sectors (i.e. business, civil society, science and government). However, there is considerably less understanding of whether the initiatives formed by local governments and diverse partnering actors actually deliver transformative capacity and how this could be developed (cf. Wilhelmer et al. 2018). Furthermore, while the transformative capacity framework significantly advances understanding into the particular sets of resources, skills and interactions that governments and other stakeholders require for propelling sustainability advancing transformations in urban settings (Wolfram 2016), empirical understanding of the extent to which municipalities demonstrate these is lacking (cf. Wolfram 2018; Ziervogel 2018). Further work in this area thus provides an opportunity to generate renewed insights into the transformative potential of the collaborative efforts of local governments to achieve sustainability transformations.

Against this backdrop, this study aims to determine the extent to which sustainability initiatives formed by local governments and diverse partnering actors around the world reflect the various components of urban transformative capacity and to identify any patterns and interrelations. The primary research question addressed is, ‘What patterns can be identified regarding the presence of individual components of transformative capacity and their interrelations with other components?’ We use Wolfram’s (2016) analysis of urban transformative capacity as an evaluative framework to carry out a secondary analysis of a database of 400 flagship sustainability initiatives from over 200 local governments (Castán Broto and Westman 2017) and examine transformative capacity in practice.

## Theoretical dimensions

### Conceptualisations of transformative capacity

Ideas of transformative capacity can be traced to the field of management science—well before this term was integrated into urban sustainability discourse. Garud and Nayyar (1994) used the term transformative capacity to describe the exploitation of innovation and knowledge generated within a company to spur technological advances, create new business opportunities and increase competitive advantage. In this sense, the term transformative capacity situates firms in the context of exogenous technological change (Pandza and Holt 2007). Garud and Nayyar (1994) argue that transformative capacity requires a long-term approach without an immediate payoff in addition to active strategies to promote change by building on previous resources (knowledge, human, technological) and experiences. As supported by sustainable transitions literature, reflective and iterative learning is integral to fostering transformative capacity (cf. Luederitz et al. 2017).

More recently, studies of transformative capacity in human organisations have embraced systems thinking from ecological sciences, emphasising the need for firms to retain competitiveness while becoming ecologically sustainable. Capra and Luigi Luisi (2014) argue that a major barrier to achieving organisational transformation is a reliance on top-down, command-and-control management approaches. This traditional and mechanistic approach positions the firm as a closed-system and ‘machine’. It places emphasis on divisions of labour, defined hierarchies of management and avenues of communication, centralised control, uniform standards and procedures, and planning rather than improvisation (Grobman 2005). Relationships with the exterior are undervalued and change is seen as a linear, predictable and controllable process that can be brought about by top-down strategies and commands. Scholars argue that efforts to transform organisations through this mechanistic paradigm are generally unsuccessful since they fail to recognise the de-centralised, nonlinear, chaotic, unpredictable and uncontrollable dynamics of transformative processes (Karp 2006). As such, these scholars have advocated for an alternative understanding of organisational change informed by insights from ecological systems. This research seeks to support deep transformations in human systems by advancing strategies that foster decentralised action and by creating ‘meaningful disturbances’ shaped by ‘guiding principles’ and shared visions and goals rather than top-down instructions (Capra and Luigi Luisi 2014, p. 318). This emerging paradigm envisages deep transformations in human systems through bottom-up changes in communities of practice that, through nonlinear feedbacks, can influence the properties of the entire organisation. Guiding principles, shared visions and goals—rather than top-down interventions—are crucial for this.

To what extent can insights from theories of transformative capacity in business organisations help us understand processes of change in cities? Evidently, organisational transformation discourse holds much relevance to urban transformative capacity with regard to understanding change as a decentralised process that is brought about from within, rather than imposed from outside. This points to the importance of social learning and collective action (Folke et al. 2005; Olsson et al. 2010; Westley et al. 2011). However, even if business organisations and cities may present similarities, several challenges arise when applying these insights from organisation management to urban contexts. For instance, one area of tension concerns the goal or purpose of the transformative process. Cities and urban areas consist of heterogeneous elements including material, natural and human systems that are shaped by diverse interests, viewpoints, agendas and activities. While groups and actors in a city can (and often do) formulate visions for change, these might not be shared by everyone. Frequently, work on sustainability transitions in cities assumes that collective visions can be shaped through collaborative envisioning-activities and that engaging far-sighted stakeholders can help engineer trajectories of urban transformation in purposeful experimentation and reflective evaluation (Nevens et al. 2013). Such propositions may downplay the particularistic and political interests inherently embedded in visions of transformation that individual actors hold (Meadowcroft 2009) in addition to the potential plurality of alternative or competing visions in particular geographies (Delina and Janetos 2018). This understanding calls for a framing of sustainability transformations in urban areas as processes following a constantly moving target in the absence of a single vision of a desirable equilibrium state (Perry 2016).

### A framework for evaluating urban transformative capacity

Wolfram (2016) identifies a set of components determining the extent of transformative capacity in urban areas that enable or drive purposive systemic change towards sustainability. As shown in Table 2, these fall into three categories: (1) agency and forms of interaction, (2) development processes and (3) relational dimensions. The first set (agency and interaction) refers to the development and application of novel governance arrangements based on broad participation, a diversity of actor networks, socially embedded leadership, and the empowerment of communities. This set of criteria draws on long-standing knowledge related to the need for governance strategies aiming to support transformative change to involve a heterogeneity of actors, interactions between multiple organisational and administrative levels, and to rely on self-organisation rather than hierarchical steering (Folke et al. 2005; Rijke et al. 2013). The criterion of multiform and inclusive governance also captures the requirement of governance arrangements seeking transformations of complex socio-ecological systems to involve negotiation and collective decision making in the orchestration of large actor networks (Olsson et al. 2006). The criteria of empowerment of communities and addressing social needs corresponds to the notion that responses to sustainability challenges provide a unique opportunity for transformative change in socio-economic and political structures and a parallel potential to address social inequalities (Agyeman 2013).

**Table 1:** Distribution of selected initiatives and cities per world region

World regions	Number of actions	Number of cities
East Asia Pacific	87	52
South Asia	33	20
Europe and Former Soviet Union	62	41
North America	58	22
Latin America and Caribbean	66	41
North Africa and Arab States	23	16
Sub-Saharan Africa	71	33
Total	400	225

The second set of criteria (development processes) refers to system awareness, collective visions, practical experimentation, reflexivity, capacity building, and institutional mainstreaming. On the one hand, insights about system awareness draw on the traditional management literature and associated insights related to strategic knowledge production required for organisational change (Garud and Nayyar 1994). On the other hand, this set of criteria reflects recognition of the need to develop knowledge that is both shared by a range of stakeholders and that creates awareness about path dependencies that may prevent transformative change (Kemp et al. 2007). The crucial role of disruptive and purposeful experimentation in processes of transformation is widely visible in scholarship in fields such as transition management (Rotmans and Kemp 2001; Rotmans and Loorbach 2009), transformations of socio-ecological systems (Olsson et al. 2010) and the introduction of novel governance strategies (Bulkeley and Castán Broto 2013).

The third set (relational dimensions) affects all other components and the levels of agency or scales where processes of transformation are realised. As such, transformative capacity extends across multiple agency levels or across geographical locations, as well as multiple domains. These criteria thus capture knowledge about the complexity of interactions involved in systems transformations. It relates to the dynamics that unfold across multiple levels of structuration in socio-technical transitions (Geels 2002, 2005), the interactions that play out between multiple domains in the transformation of socio-ecological systems (Westley et al. 2011), and the interactions that stretch across geographical scales and administrative borders in multilevel governance processes (Hooghe and Marks 2001; Betsill and Bulkeley 2004; Bulkeley and Betsill 2005).

The collective ability of actors to realise change relates to opportunities for either channelling resources to specific development processes or altering the rules that govern such resources. Through this definition, Wolfram (2016) directs attention not only to current access to resources but also to latent strengths or abilities to pursue transformative change. Transformations require the combined recognition of place-specific capacities with cross-scale relations. That is, while certain dimensions of transformative capacity are fundamentally place-based (e.g. attention to societal needs and practical experimentation in a particular locale), others are dependent on connections that extend beyond that specific geography (e.g. actor networks and infrastructure systems). Therefore, in socio-ecological systems, both individuals or closed groups can create meaningful disturbances and render change possible (Westley et al. 2013).

Given the mounting urgency of the global imperative to radically transform urban settlements into drivers of rather than obstacles to advancing sustainability, operationalising Wolfram’s (2016) framework of indicators for transformative capacity provides an important opportunity for both practitioners and scholars to identify the weaknesses and strengths of sustainability initiatives that either enable or limit processes of change. While much scholarship examines sustainability initiatives driven by ambitions to transform urban systems and advance urban sustainability (Bulkeley and Castán Broto 2013; Trencher et al. 2014), this evaluative framework provides a so far unrealised opportunity for self-reflection and reiterative learning to improve the transformative potential of these collective efforts.

## Methodology

We examine an existing database of 400 initiatives from 225 cities to systematically determine the extent to which the various components of transformative capacity are exhibited in efforts to advance urban sustainability around the world (Table 1). A ‘sustainability initiative’ refers to an action (i.e. a project, policy or incentive) either led by or involving the local government with an explicit objective to address sustainability in a particular locale. The database was developed by Castán Broto and Westman (2017) to determine sustainability governance trends.

**Table 2:** Operationalisation of transformative capacity criteria

Criterion or sub-criteria	Satisfied when evidence (i.e. explicit references to) found for…	Explanation or exceptions The criterion was deemed satisfied when evidence found for *either of* the following indicators
Inclusive, multiform urban governance (C1)		
Participation/inclusiveness (C1.1)	Citizens and/or civil society organisations participating directly in planning and/or decision-making processes	• Active participation of citizens and/or civil society organisations in decision-making processes through mechanisms such as participatory or collaborative planning, public consultation, or initiatives led by communities • We excluded initiatives where public actors participated in the activities of an NGO but without formal involvement of a public agency, as well as initiatives with references to intentions of ‘public involvement’ without specific details on how this was realised
Diverse governance modes/networks (C1.2)	Different and various stakeholders working together and building connections between sectors in different manners	• A variety of actors involved throughout the initiative • Institutional development and capacity building in relation to building social capital, for example, by creating relations of trust between stakeholders or formal communication channels for marginalised groups that could facilitate future collaboration
Sustained intermediaries and hybridisation (C1.3)	An intermediary positioned between the stakeholders of a project	• Involvement of external actor (individual or organisation), such as an NGO or a consultant that are not themselves direct stakeholders (such as a local inhabitant, or a government representative) • Measures to facilitate sustained involvement of the intermediaries such as independent streams of funding or integration into project governance structures
Transformative leadership (C2)	Leadership acting as a driving collaborative force in an initiative	• Leadership linking local action with global arenas and processes • Certain actors championing a case and inspiring enthusiasm through articulation of shared values or visions
Empowered communities (C3)		
Social needs (C3.1)	Either analysing or addressing social needs	• Explicit references to local social agendas, in particular those that addressed vulnerable groups and issues of social marginalisation • Strategies seeking to improve the wellbeing and quality of life of urban citizens that also pay particular attention to questions of social justice
Autonomous communities (C3.2)	Integrating into the design of the project different aspects of community empowerment	• Project design providing citizens/communities not only with new or improved facilities and/or services, but also with new skills, training and abilities, improved access to political processes, greater independence and self-efficacy • Measures or resources to allow direct involvement of communities in decision making processes or independently realise their objectives
System awareness (C4)		
Baseline analysis and system(s) awareness (C4.1)	Agendas aiming to tackle sustainability challenges after deliberate analysis of urban systems	• Actively analysing existing governance structures, institutional landscapes and natural resource conditions and efforts to use this knowledge to plan interventions • Strategic knowledge management processes carried out to understand current conditions
Recognition of path dependencies (C4.2)	Explicitly tackling systemic barriers to change	• Recognising systemic barriers (including regulations, physical barriers, but also cultural values, such as perceptions or established routines) that need to be overcome for the project to become viable or successful • Explicit strategies to overcome these path dependencies
Foresight (C5)		
Co-production of knowledge (C5.1)	Involvement of various and multiple stakeholders in knowledge production processes	• Involvement of diverse groups such as experts, external stakeholders, civil society and other government authorities in production of knowledge related to the targeted sustainability issues
Collective vision for change (C5.2)	An explicit future vision shared among stakeholders as a means for motivating partners and fostering commitments	• Visions that are at once (1) explicitly formulated, (2) aiming for radical change, and (3) supported by a wide range of stakeholders • We excluded initiatives based on visions that were not radical (e.g. incremental agendas such as air pollution protection) or that appeared to be endorsed be few actors only (such as political elites)
Alternative scenarios, future pathways (C5.3)	Comparative scenarios that evaluate the mutual shaping of social, ecological, economic and technological dimensions	• Evaluation of multiple alternative visions or pathways for change • This includes, for example, envisioning exercises that take into account different possible development outcomes associated with different policies or environmental conditions
Experimentation with disruptive solutions (C6)	Deliberate use of experiments or ideas that seek to challenge the existing landscape of established policies, technologies or social practices	• Active trialling or demonstration of new solutions (i.e. both social practices as well as new technologies) as opposed to plans or intentions to support new solutions) • New solutions were radically different from existing practices or technologies • Incremental improvements, such as upgrades of existing equipment to improve energy efficiency, were not included
Innovation embedding (C7)		
Resources for capacity development (C7.1)	Project stakeholders sharing resources for capacity development outside the project to disseminate and multiply results	• Sharing of lessons learnt, knowledge and expertise through events, workshops, publications (printed or online) or offering direct advice and support to groups that could benefit from the expertise • Provision of resources for training of individuals or organisations, or partnering up with new organisations and providing funding to share insights gained through the initiative
Mainstreaming transformative action (C7.2)	Attempts to generalise the project operation or results beyond the initial context of application	• Replicating or applying the project itself or various processes, methods, components or solutions in different settings and locations • Embedding changes in institutional settings to allow for the continuity of the project and lessons learnt as ‘the new norm’
Regulatory frameworks (C7.3)	New regulation was established as a result of the project or as part of the project activities	• Projects leading to lasting change through embedment in legal, regulatory and policy frameworks • For example, this included cases where initiatives led by communities or civil society cause local authorities to alter policy or regulation, or where local regulation is absorbed on a national level
Reflexivity and social learning (C8)	Stakeholders reflecting on learning and capacity building processes	• Procedures for recording, evaluating and assessing procedures • Reflecting on how the project unfolded as well as if and how the project visions were met. This includes references to open discussions and critical dialogue on project development as well formal methods for reporting and monitoring
Working across human agency levels (C9)	Project activities contributing to capacity development across human agency levels	• Capacity building activities occurring across agency levels including individuals, households, social groups, organisations, networks and society (collaboration across either of these levels was considered sufficient evidence to meet this criterion)
Working across levels and scales (C10)	Project activities contributing to building capacity across geographical or political–administrative levels	• We considered whether initiatives involved capacity building at different levels of government, including local, municipal, regional, national and supranational (collaboration across either of these scales was considered sufficient evidence to meet this criterion)

The original database data was created during 2015 and 2016. An internet search of secondary and grey literature (government reports, project materials, websites) helped to identify at least one or more ‘flagship’ initiatives in each city, i.e. those initiatives that key actors present as significant and representative of the sustainability approach in that city. Around half of the initiatives in this sample are led by local government agencies while the other half is led by other actors (in industry, academia, civic organisations or other government bodies)—often in partnership with local governments. The information was coded in a database structured around variables such as the initiative’s name, location, key actors, implementation dates, objectives and outcomes. Coding also characterised the type of initiative (sectors included air pollution/climate change, ecological protection/biodiversity, energy, housing, land use/planning, sanitation/water, transport, urban greening/urban agriculture and waste), and the type of governance arrangement (i.e. the leader of the initiative and the presence of any partnership). The majority of initiatives target water and sanitation sectors—reflecting an emphasis on traditional development concerns in large parts of the world. Conversely, another large set of initiatives deals with environmental challenges typical of the global North such as waste reduction, energy management and climate change mitigation.

Building on this original database, we reviewed each entry from the perspective of the ten indicators in Wolfram’s (2016) criteria for urban transformative capacity. Relevant data analysis and coding took place between January and February 2017. For each initiative we reviewed the original data sources for evidence to support a judgement about whether a given capacity component was present or not. This process involved adding new data to record the extent to which each initiative met the urban transformative capacity criteria. Table 2 explains how we developed and applied specific rules for the operationalisation of each criterion and sub-criteria. In addition to the qualitative analysis of the results reported in our findings, we also performed a quantitative analysis to determine whether actor constellations were associated with specific governance trends. This was carried out by creating cross-tabulations between the different components and testing for correlations between variables through a *χ*^2^ test.

## Results

**Fig. 1:**
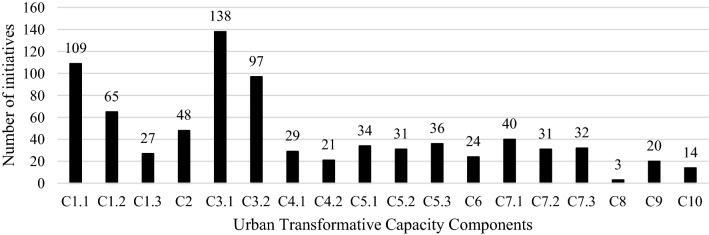
Share of initiatives that satisfied the criteria for transformative capacity (*n* = 400)

Aggregate results of our database analysis are summarised in Fig. 1 and Table 3, while the following sections unpack the most significant findings. Figure 1 demonstrates that overall criteria fulfilment is very low. Only five criteria were met by more than 44 initiatives: C3.1 (satisfied by 35% of initiatives), C1.1 (27%), C3.2 (24%), C1.2 (16%), and C2 (12%). All the other criteria were satisfied in only less than 10% of the initiatives. The least frequently satisfied criteria by far is reflexivity and social learning (C8), which was met by 0.75% of initiatives in addition to the two criteria assessing relational dimensions, namely building capacities across human agency levels and scales (C9 and C10, satisfied by 5 and 3.5% of initiatives, respectively).

**Table 3:** Number of initiatives satisfying TC criteria per sector

	C1	C2	C3	C4	C5	C6	C7	C8	C9	C10	Total (*n*)	Total (%)
Air/climate change	16	6	14	7	13	1	6	1	2	3	36	9
Eco-city/eco-business	5	3	2	1	8	2	6	0	0	0	19	5
Eco-protection/biodiversity	9	6	5	3	9	1	9	0	0	2	25	6
Energy	9	5	12	0	4	1	11	0	0	0	48	12
Housing	42	10	55	13	19	9	31	2	6	2	35	9
Land use/planning	54	8	39	11	26	3	19	0	7	1	57	14
Sanitation/water	37	4	51	9	13	2	16	0	2	4	66	17
Transport	4	0	9	1	1	1	0	0	0	0	38	10
Urban greening	13	3	26	4	6	3	8	0	2	0	29	7
Waste	12	3	22	1	2	1	2	0	1	2	47	12

Table 3 shows the number of initiatives meeting the various criteria for transformative capacity in each sector. The largest number of initiatives exhibiting evidence of the criteria emerged in the housing and land-use sectors, followed by sanitation. Conversely, initiatives in the transport, eco-city and waste sectors present the least evidence for meeting the criteria.

Table 4 presents an overview of the Pearson *χ*^2^ coefficients to analyse the independence between variables. Only 48 tests (31%) are valid. The table shows high levels of correlation between variables. For multiple criteria (C1.3, C4.2, C6, C8–10) the number of positive cases was so low that the majority of tests are not valid.

**Table 4:** Independence tests between the different TC criteria

	C1.1	C1.2	C1.3	C2	C3.1	C3.2	C4.1	C4.2	C5.1
C1.1	X	122.016	37.289	51.264	33.211	68.411	48.597	26.778	30.588
C1.2	X	X	46.425*	57.621	37.839	53.999	55.765*	41.395*	13.197
C1.3	X	X	X	35.828*	37.903	51.629	29.295	45.906*	11.305*
C2	X	X	X	X	11.419	30.406	25.557*	42.771*	36.298*
C3.1	X	X	X	X	X	108.963	19.889	21.164	9.729
C3.2	X	X	X	X	X	X	28.987	38.793	20.241
C4.1	X	X	X	X	X	X	X	31.359*	9.831*
C4.2	X	X	X	X	X	X	X	X	11.480*
C5.1	X	X	X	X	X	X	X	X	X
C5.2	X	X	X	X	X	X	X	X	X
C5.3	X	X	X	X	X	X	X	X	X
C6	X	X	X	X	X	X	X	X	X
C7.1	X	X	X	X	X	X	X	X	X
C7.2	X	X	X	X	X	X	X	X	X
C7.3	X	X	X	X	X	X	X	X	X
C8	X	X	X	X	X	X	X	X	X
C9	X	X	X	X	X	X	X	X	X
C10	X	X	X	X	X	X	X	X	X

	C5.2	C5.3	C6	C7.1	C7.2	C7.3	C8	C9	C 10
C1.1	37.355	22.880	20.010	20.515	37.355	45.522	8.070*	41.815	19.275*
C1.2	43.173	14.898	33.225*	18.421	50.091	77.653*	15.578*	53.393*	32.457*
C1.3	8.482*	10.128*	28.665*	38.169*	13.380*	14.170*	17.240*	62.564*	10.975*
C2	87.764*	27.086*	51.905*	39.152*	67.525*	36.910*	8.554*	56.001*	19.838*
C3.1	6.152	7.761	26.944	10.404	19.777	18.087	5.739*	28.697	12.470*
C3.2	17.117	14.280	35.798	50.641	25.099	22.565	9.442*	57.367*	17.579*
C4.1	31.254*	8.748*	18.238*	20.824*	60.123*	51.731*	15.869*	24.109*	39.429*
C4.2	28.547*	22.909*	68.068*	26.586*	20.290*	29.905*	22.921*	9.208*	41.248*
C5.1	31.460*	9.578*	14.021*	32.915*	12.941*	9.175*	2.397*	26.859*	7.515*
C5.2	X	22.195*	41.081*	13.525*	65.786*	47.181*	0.254*	14.578*	8.797*
C5.3	X	X	25.321*	6.566*	11.589*	2.328*	12.273*	17.377*	2.736*
C6	X	X	X	45.390*	23.374*	24.561*	4.004*	21.501*	1.766*
C7.1	X	X	X	X	38.080*	32.432*	1.829*	47.368*	5.560*
C7.2	X	X	X	X	X	47.181*	2.767*	52.564*	15.869*
C7.3	X	X	X	X	X	X	15.253*	15.363*	16.647*
C8	X	X	X	X	X	X	X	24.199*	7.965*
C9	X	X	X	X	X	X	X	X	8.243*
C10	X	X	X	X	X	X	X	X	X

In every case, df = 1. Critical value 7.879 for a *p* = 0.005; critical value 10.828 for a *p* = 0.001

*One or more cells have less than 5 (test is not valid)

### Inclusive and multiform urban governance

Evidence of inclusive and multiform urban governance (C1) varied considerably. Inclusion and participation (C1.1) was met in a large share of the initiatives: 109 of the 400 cases (27%). Around a fourth of the initiatives involved direct participation of citizens, communities or civil society organisations. These initiatives ranged from ones that strongly satisfy the criteria (such as the Participatory Budgeting Project in Lisbon, which attempts to change the nature of governance through processes of collective decision making around public expenditure, to cases exhibiting weaker evidence (for example an initiative in Austin, Texas, US, where an online platform allowed citizens to share comments and ideas about urban transport or the Kuala Lumpur Structure Plan 2020, which compiled and reflected the results of hundreds of objections to the Plan). The database also contains examples of how formerly excluded stakeholders, such as landless shack dwellers, homeless women and ethnic minorities, were actively involved in project development. Most of the initiatives meeting this criterion emerged in the sectors of land use and planning (53%) and housing (49%). In contrast, sectors characterised by large-scale infrastructure investments involve participation less frequently. C1.1 was strongly correlated with all the other criteria for which the test was valid.

Evidence of governance networks and cross-sector interaction (C1.2) was identified in a smaller number of initiatives (65 out of 400 or 16%). These were also most common in housing and land-use projects with a pro-poor profile and were least common in the transport and waste sectors. This suggests a synergy between a range of strategies that seek to build inclusive and multiform urban governance. C1.2 was also correlated with all the other sub-criterion for which the test was valid. Evidence of the third sub-criterion (C1.3, sustained intermediaries and hybridisation) was only identified in 27 initiatives (7%). Initiatives that involve intermediaries are somewhat more diverse. Intermediaries are often either civil society organisations operating as advisors and to facilitate communication between stakeholders or expert organisations such as consultancy firms or international banks providing technical advice. For example, Future Generations (an intermediary NGO) was involved in setting up a participatory budgeting system in Huanucu, Peru (Altobelli 2008). The correlations suggest that intermediation is a crucial factor for empowered communities (C3), including social needs (C3.1) and autonomous communities (C3.2). Intermediation is also a critical factor in building system awareness.

### Transformative leadership

The criterion of transformative leadership (C2) was satisfied in only 48 initiatives (12%), with the sectoral distribution not appearing to follow any clear trend. This criterion is strongly connected to the presence of identifiable actors providing leadership and processes of leadership development. In Cochabamba in Bolivia, for example, the María Auxiliadora community has advanced an agenda of empowerment that has created new roles for citizens in the local economy and political system (World Habitat 2017). The Odisha Alliance, in Bhubaneswar, India is a multi-institutional partnership that mobilised 10 000 members to secure housing and infrastructure for the urban poor. Both initiatives build upon transformative, issue-focused leadership. The María Auxiliadora community is led by an elected neighbourhood committee in which, based on traditional Andean leadership structures, the presidency and vice-presidency must rotate every 2 years for each family to gain experience in leadership. Trained local leaders now organise and take part in events around the right to housing, presenting their particular experiences to NGOs, universities and other community groups, as well as media. The Odisha Alliance is facilitated by a partnership of a non-profit organisation (Urban and Development Resource Centre) and two networks of community organisations (the women’s organisation Mahila Milan, and the Odisha/National Slum Dwellers’ Federation). There was a strong correlation between the criterion C2 for transformative leadership, criterion C3.1 for social needs and C3.2 for autonomous communities, thus reflecting the interconnection between transformative leadership and mechanisms that empower communities.

### Empowered and autonomous communities of practice

The sub-criterion of addressing social needs (C3.1) was met in 138 initiatives (35%). Many meeting this sub-criterion have a traditional development profile (e.g. providing sanitation, water, and housing for the urban poor) given explicitly goals to address social needs. In this case, we could rule out the independence of variables between C3.1 and C5.2 (collective vision for change) and C5.3 (alternative scenarios, future pathways). This may reflect that social needs may relate to intra-generational solidarity and may not always be oriented towards building foresight, as argued by participatory planning scholars (e.g. Castán Broto et al. 2015). Additionally, there is some evidence—albeit weaker—of marginalised social needs articulated in broader public policy and urban plans. As much as 86% of initiatives in the housing sector and 50% of those related to water and sanitation met this criterion, and the majority were carried out in the urban global South.

The sub-criterion of community empowerment and autonomy (C3.2) was met in 97 initiatives (24%). These often concerned the mobilisation of residents in informal settlements with the goal of improving local living conditions. As a result, most lie in the housing sector (where 71% satisfy the criterion) and are located in the global South. Examples of projects that put communities at the forefront of the planning, design and management processes range from housing projects, such as the Housing Cooperative UFAMA al SUR Montevideo in Uruguay (World Habitat 2017), to programmes for the mobilisation of social groups for environmental action, such as the movement of waste collectors ReviraVolta Expocatadores formed in the CRUMA Cooperative in Sao Paulo, Brazil and which plays a crucial role in waste management (Ponce 2012). This criterion was also correlated with all the others for which the test was valid, highlighting the importance of including communities as autonomous actors at the outset of projects.

### System(s) awareness and memory

Evidence for the sub-criterion of baseline analysis and system awareness (C4.1) was rare and only identified in 29 cases (7%). The reason for this is that few initiatives explicitly frame their activities in relation to existing structures, processes or resources in which they operate. Those satisfying this criterion either involved resource mapping elements or, less often, explicit efforts to develop new institutional structures by building on experiences over time. An example of the former is the establishment of an inventory of parks and open space in Washington, DC (NCPC 2010) called CapitalSpace. This initiative began by completing the first comprehensive analysis of Washington’s parks and open space in nearly 40 years. Meeting both local and national needs from Washington’s park system required considering the wide variety of park types, sizes and traits, coupled with shared jurisdiction between local and federal authorities. The second sub-criteria related to system awareness and recognition of path dependencies (C4.2) was satisfied in only 21 initiatives. These involved explicit recognition of systemic barriers to change and associated strategies for overcoming these, concerning for instance current regulations, problematic infrastructure or cultural values such as established routines and patterns of thinking.

### Urban sustainability foresight

The first sub-criterion for sustainability foresight, diversity and transdisciplinary co-production of knowledge (C5.1) was met in only 34 initiatives (8.5%). This criterion was most often met in participatory initiatives in the housing and land-use sectors, out of which the majority were related to tackling poverty issues. The second sub-criterion, collective vision for sustainability changes (C5.2), was similarly scarce and met in only 31 initiatives (7.7%). Many of these emerged in the housing and land-use sectors, usually in projects where communities and neighbourhoods adopted a specific development vision for themselves. Attribution of the third sub-criterion, envisioning alternative scenarios and future pathways (C5.3), was closely related to the formulation of visions that project actors framed as radical or that represented significant departures from the norm. Evidence of this was observed in 36 cases (9%). Initiatives meeting this criterion also tend to emerge in relation to land-use planning (17%) and eco-city projects (21%) where development visions for sustainability—and strategies for reaching these—are explicitly articulated. This criterion is also often met by city greening strategies (17%) and municipal climate mitigation or adaptation plans (17%), which tend to include formal development visions and policy instruments used to reach these (e.g. predictions for future carbon emission reductions under different policy strategies) (cf. Glaas et al. 2018).

### Diverse community-based experimentation with disruptive solutions

The criterion of community-based experimentation (C6) was satisfied in 24 initiatives (6%). The majority of these occurred in the housing sector (where 26% of initiatives satisfied the criterion) and involved community-based efforts to build sustainable practices. An example is a self-managed vertical housing project in Sao Paulo, Brazil (World Habitat 2017). This initiative is based on the concept of ‘mutual help construction’ of multistorey buildings for densely populated urban areas and integrates homeless groups and social movements into decision-making processes at all stages. Use of mixed construction technologies, with steel stair towers and structural blocks, make possible the construction of vertical buildings through mutual help, with unspecialised labour. This is further facilitated through community management of the process, including establishment and management of work teams, sub-committees, accident prevention, kitchens and day nurseries. Experimentation occurs at many levels, reimagining both the physical structure of the built environment and the network of institutional support.

### Innovation embedding and coupling

Evidence of transformative capacity related to access to resources for capacity development (C7.1) was also low and identified in 40 cases (10%). This component was most often found in projects where stakeholders shared resources for capacity development to disseminate and multiply the results of the project. The sub-criterion of planning and mainstreaming for transformative action (C7.2) was met in 32 initiatives (7.7%). This was satisfied where there was evidence of attempts to mainstream the project or its results by producing a methodology that could be applied in different settings and locations, by embedding changes in institutional settings, or by establishing the activities of the project or lessons learnt as ‘the new norm’. Compelling examples of innovation mainstreaming are initiatives where the effectiveness of an approach is demonstrated to public authorities, and the new practice is absorbed into institutional guidelines and practices. Most initiatives of this kind occurred in the form of novel procedures related to local housing strategies (26% of initiatives in this sector met the criterion). The Project Liter of Light in Manila, Philippines (World Habitat 2017) is one example. This aims to provide high-quality lighting to poor communities and was conceived as an open-source, ‘do-it-yourself’ (DIY) programme replicable by anyone around the world using readily available materials and basic electronics skills. It included both an evaluation of resources available and an attempt to facilitate mainstreaming through provision of detailed online tutorials on required materials and step-by-step installation procedures. The final sub-criterion for innovation embedding, reflexive and supportive regulatory frameworks (C7.3), was met in 30 initiatives (8%), mainly exhibited in cases where project outcomes result in new regulation. The conceptual proximity to the previous sub-criterion produces a similar distribution across sectors. That is, most were in the housing sector (where 29% of initiatives meet the criterion), with many satisfying this criterion also meeting C7.2.

### Reflexivity and social learning

The criterion of reflexivity and social learning (C8) was, by far, the least frequently observed—with only three cases that satisfied this. This result suggests either that few projects involve explicit attempts to reflect on insights and shape activities according to how previous processes have unfolded, or that, because of the vagaries of the project cycle, most initiatives rarely report such processes of learning. The sole three initiatives meeting this criterion also meet most of the other criteria. Hence, reflexivity and social learning may be a crucial indicator of progressive actions for sustainability transformations in the sense that, if an initiative is found meeting this indicator, it appears likely to satisfy the other capacities. Having said this, with only three positive cases we could not statistically evaluate with the *χ*^2^ test the degree to which C8 related to other criteria.

### Working across human agency levels

The criterion of working across human agency levels (C9) was met in 20 initiatives (5%), in those that involved activities contributing to capacity development across human agency levels (individuals, households, social groups, organisations, networks and society). Distribution of this criteria mostly mirrors that of inclusive and multiform governance processes (most common in housing and land-use projects in the global South) due to explicit efforts to create activities and collaboration across sectors. For example, the Dajopen Waste Management (DWM) Project in Kitale, Kenya (UN-Habitat 2011) involved 21 000 people in waste management and organic farming, eight community groups in producing a range of recycled products, and numerous policy makers, government officials, students and non-governmental institutions.

### Working across political–administrative levels and geographical scale

We also observed a widespread absence of the last transformative capacity component, working across levels and scales (C10). This was met in only 14 initiatives (3.5%), the second lowest number in our study. This criterion was deemed satisfied wherever there was explicit mention of project activities contributing to building capacity across geographical or political–administrative levels (cf. Borgström et al. 2018). The sectoral distribution for this criterion is similar to institutional embedding and most commonly met in the housing and land-use sectors. In Santos, Brazil, the Associação Cortiços do Centro, Condomínio Vanguarda created the grassroots pressures that led to the ‘Concessão de Direito Real de Uso’ (i.e. Concession of full rights to the use of land) which allows federally owned land to be handed over to the community for social housing purposes. This initiative has unfolded through collaboration across the local, state and federal levels to secure necessary land and funding.

## Discussion and conclusions

This study aimed to determine the extent to which sustainability initiatives formed by local governments and diverse partnering actors around the world reflect the various components of urban transformative capacity and to identify any patterns and interrelations regarding their presence or absence. The systematic analysis of the database shows that despite an explicit orientation towards change for sustainability, transformative capacity is far from ubiquitous. Even the two most widely satisfied criteria—participation/inclusiveness and social needs—were only observed in about a third of the surveyed initiatives. Other criteria were rarely met. Among others, there was only occasional evidence of system awareness, especially with regard to recognition of path dependencies, reflexivity and social learning working across human agency levels, and working across levels and scales. Evidence of transformative capacity tended to feature more prominently in housing, land-use planning and, to a certain extent, water and sanitation initiatives. In contrast, transformative capacity was atypical in energy and transport initiatives, as well as in initiatives related to the construction of eco-city and eco-businesses. Findings suggest that transformative capacity appears to emerge in relation to wider processes of institutional- and social-learning since evidence of such processes was often found in initiatives that linked outcomes to a city-wide vision of planning and development. In contrast, transformative capacity is rare in sectors dominated by techno-efficiency and economic discourses. The widespread lack of evidence for the various components that form transformative capacity raises concerns that this set of transformative attributes is in fact rarely found in sustainability action on the ground.

The analysis demonstrates the multidimensional nature of transformative capacity. This was confirmed empirically by the high levels of correlation observed between different criteria. Even when the statistical test was not valid, a qualitative examination suggests that initiatives satisfying the most rarely identified criteria (C8–10 concerning social learning, reflexivity, and working across agency and geographical scales) tend to be those that already meet several other criteria. For example, the criterion reflexivity and social learning (C8) was only met in three initiatives. Closer examination revealed these three cases met several other criteria simultaneously, suggesting a lesson from this overlap. Initiatives that displayed a high degree of reflexivity and social learning automatically considered inclusive and multiform forms of urban governance, deliberately tried to empower communities, exhibited foresight practices, and furthermore, worked across levels of human agency and scales. While this suggests that a component such as reflexivity and social learning may be a useful indicator of initiatives with a higher ability to deliver urban transformations, more information is needed on initiatives that explicitly address that component. This finding aligns with several other works that point towards the centrality of social learning processes to efforts to carry out urban transformations (Bos et al. 2013; Mieg and Töpfer 2013; Nevens et al. 2013; Castán Broto et al. 2014; Castán Broto and Dewberry 2016). In contrast, the results show weak evidence of the role of intermediaries, despite being prominent actors in transition studies (Hamann and April 2013).

In this context, the transformative capacity framework offers a new vocabulary to understand the process of urban sustainability transformation. It supports the systematic analysis of different dimensions of transformative capacity as a means to develop alternative forms of evaluation of projects and programmes that aim to achieve sustainability transformations in urban areas. If applied for evaluative or self-reflection purposes by a team of project actors, the framework could provide an opportunity to identify weaknesses, strengths, and opportunities to realise more effective transformative action for urban sustainability goals. In this way, our study contributes to growing interest in literature around how more qualitative types of outcomes from urban sustainability initiatives can be measured (Luederitz et al. 2017).

However, the breadth of this framework and sheer volume of indicators, coupled with our results, suggest that it might be unreasonable to expect all urban actors and initiatives to explicitly and successfully address all components simultaneously. Sanchez Rodriguez et al. (2018) raise similar concerns regarding the UN Sustainable Development Goals. They argue that the sheer number of goals and targets risks creating confusion or competing agendas for policy makers, drawing attention to the need for multidimensional approaches and awareness of interaction amongst goals. Heeding this, differing local circumstances, objectives and stakeholder capacities might therefore require various sustainability initiatives to play to innate strengths and pursue components with the most relevance or importance as a pre-condition for others. Our findings suggest that strategies prioritising social learning and reflexive action may be a means to foster other components of transformative capacity.
